# Efficacy of Adding Bortezomib to Salvage Chemotherapy in Relapsed/refractory Acute Myeloid Leukemia a Prospective Non-Interventional Study

**DOI:** 10.22037/ijpr.2021.115475.15409

**Published:** 2021

**Authors:** Mojtaba Ghadiany, Mehdi Tabarraei, Beehnaz Varaminian, Sina Salari

**Affiliations:** a *Department of Hematology and Oncology, Faculty of Medicine, Shahid Beheshti University of Medical Sciences, Tehran, Iran. *; b *Department of Medical Oncology and Hematology, Iran University, Faculty of Medicine Tehran, Iran.*

**Keywords:** AML, Bortezomib, Salvage therapy, Refractory, Leukemia

## Abstract

Relapsed/refractory acute myeloid leukemia (RR-AML) are important types of hematological malignancy for which no effective salvage chemotherapy has been approved. The main purpose of this study was to determine the effectiveness of adding Bortezomib to salvage chemotherapy protocol in RR-AML patients. In this prospective non-interventional study, 40 consecutive patients with RR-AML attending Taleghani Hospital who underwent salvage therapy, were enrolled and subdivided into salvage chemotherapy plus Bortezomib and salvage chemotherapy without Bortezomib and the therapeutic response, adverse effects, and survival among them were determined. The results in this study demonstrated that complete response was present in 30% and 25% in Bortezomib group and the other group, respectively and the partial response was seen in 35% and 50% and no response was present in 35% and 25%, respectively (*P* = 0.621). Furthermore, in each group, 15% had a side effect (*P* = 1.000). Mean survival was 8.2 and 7.1 in Bortezomib and the other group, respectively *(P* = 0.275). Based on the obtained results, it may be concluded that adding Bortezomib to salvage chemotherapy is feasible in RR-AML patients. For evaluation of efficacy further study is recommended.

## Introduction

Acute myeloid leukemia (AML) is a common hematological malignancy leading to 20,000 new patients and 10,000 fatal cases annually (1). Despite 60-90 percent therapeutic response, the final rate of resistant/recurrent cases is high (2). The prognosis in such patients is poor (3) and these are usually considered for salvage regimens with approximately 30 to 65 percent rate of efficacy and mortality rate of 7 to 20 percent (4, 5). 

Bortezomib is a proteasome inhibiting drug used for the treatment of multiple myeloma and some lymphoma subtypes and the addition of this drug to some salvage regimens is a novel idea with promising results (1). Complementary use of this drug has also been demonstrated to be safe in these patients (3). However, some studies have shown therapeutic adverse effects (2). Combination therapy with Bortezomib plus MEC has been determined to be very effective and safe in small-sample case series (4). Since there are no standard salvage regimens for cases with relapsed refractory acute myeloid leukemia (RR-AML) many different regimens are assessed in such cases and comparisons of these regimens are important to select the best therapeutic options with the least adverse effects and highest safety profile (6-11). Hence this study was carried out to determine the efficacy and safety of adding Bortezomib to salvage protocol in RR-AML patients. 

## Experimental

In this prospective non-interventional study, 40 consecutive patients with RR-AML attending Taleghani Hospital in Tehran, Iran from December 2018 to March 2020 who underwent salvage therapy were enrolled. Inclusion criteria included RR-AML, optimal executive function, and normal hepatic and renal function. The cases with inappropriate executive function, or with severe background disease/infection were excluded from the study. 

The study was approved by the Ethical Committee in Shahid Beheshti University of Medical Sciences Subjects had been treated by their physicians. Therefore, if their physician had chosen to add Bortezomib to salvage therapy the subjects were included in the Bortezomib group; and if not, they were considered as other treatment groups (*i.e. *Flag and EMA). The background and demographic data were recorded in the checklists by clinical and laboratory assessments and interviews. The therapeutic response, adverse effects, and survival rates among them were determined and compared across the groups. 

Data analysis was carried out by statistical package for social sciences (SPSS) version 13.0. The used tests were Ch0-Square, Fisher, Independent-Sample-T, and Kaplan-Meyer analyses. P-values less than 0.05 were considered statistically significant. 

## Results

The mean age was 48.5 ± 8.9 and 50.5 ± 9.5 years in Bortezomib and the other treatment group, respectively (P = 0.508). The patients were female in 65% and 60% of cases in Bortezomib and the other treatment group, respectively (*P* = 0.744). 

**Figure 1:**
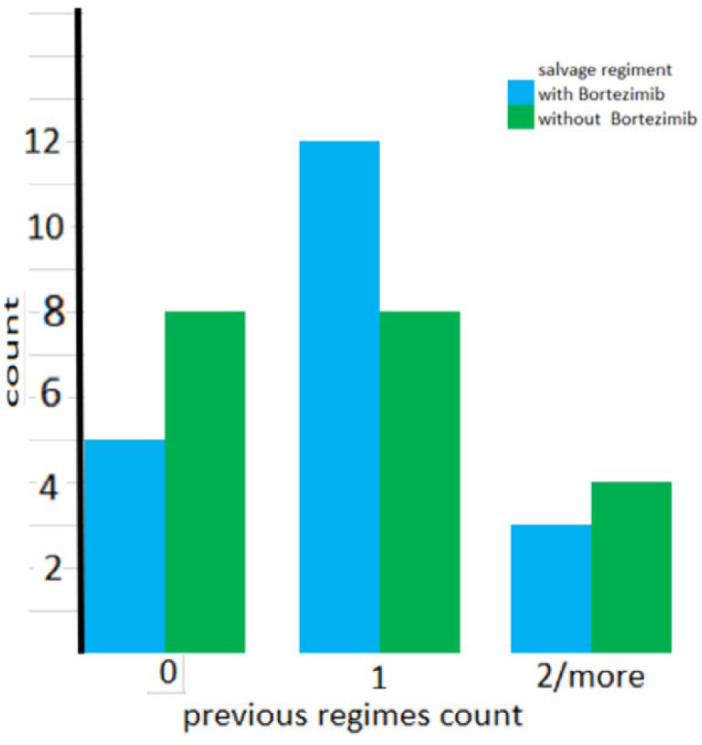
Number of previous chemotherapy

**Figure 2:**
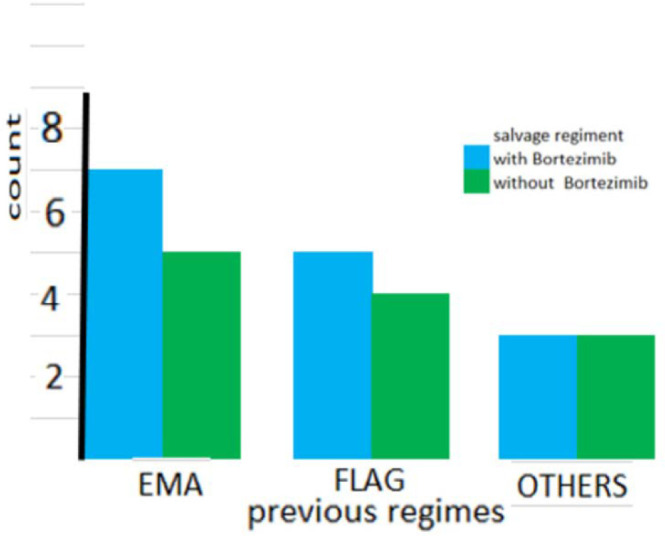
Name of previous chemotherapy

As shown in Figure 1 the number of previous salvage regimens was not significantly different between the two groups. (*P* = 0.441). Also as shown in Figure 2, the types of previous salvage were the same across the groups (*P* = 0.945). 

The relapse was more than six times in 60% and 45% in Bortezomib and the other treatment group, respectively (*P* = 0.625). The complete response was present in 30% and 25% in Bortezomib and the other group, respectively, and the partial response was seen in 35% and 50%, and no response was present in 35% and 25%, respectively (*P* = 0.621). Also, in each group, 15% had side effects (P = 1.000). Mean survival was 8.2 and 7.1 in Bortezomib and the other group, respectively (*P* = 0.275). 

## Discussion

In this study, the efficacy and safety of bortezomib and other salvage regimens were compared in RR-AML cases. The cases with RR-AML have usually poor prognosis and adverse therapeutic outcomes. Recognition of the best therapeutic outcomes is important in such cases. The partial/complete response was seen in 65 and 75 percent of patients in Bortezomib and the other salvage regimens, respectively in our study, which showed no statistically significant difference across the groups; and also, the survival and safety profile were the same in both groups. 

Walker *et al. *(7) have reported complete response in MEC plus bortezomib treatment in 56.5%, and also the total response rate of 82.5%. But the authors reported poor safety outcomes. In our study, the total response rate was 65 percent but the adverse effects were seen in a low number of the cases.

However, totally in their study, it was claimed that there is good efficacy of MEC plus Bortezomib in the AML patients. 

Advani *et al.* (6) reported the cytogenetic side effects in 19 cases with RR-AML and also reported 58 percent of the patients had poor-risk molecular mutations. However, there was no dose-response association in their study. In total four cases had died, including 1 case during treatment and three subjects by induction. Fever, neutropenia, metabolic adverse effects, gastrointestinal and skin side effects were among the reported adverse effects in patients. Our study was also successful, and the rate of efficacy and safety were the same between the two groups. 

Udvardy *et al.* (8) assessed the effects of the addition of Bortezomib to other salvage therapy regimens in 27 cases and found that these combinations resulted in high safety and low mortality rate in the induction phase. Bleeding, sepsis and neutropenia were the observed side effects in cases without pulmonary syndrome and they reported no difference with induction regimen as seen in our study for comparison of the groups. 

Attar *et al.* showed that among 30 cases with AML, the addition of Bortezomib to induction therapy regimen could result in complete response in 58 percent of cases. Only four cases were reported to have no response; but, as a whole, the safety profile was good. Their findings were totally in line with our findings. 

Based on our obtained results, it may be concluded that the efficacy of salvage chemotherapy plus Bortezomib protocol in RR-AML patients is relatively optimal and the use of these regimens is recommended. The main limitations in our study were a lack of follow-up cases and also sampling from only one center. Further studies with a larger sample population and multi-center samplings are required to attain more definite comparable results. 

**Table 1:** Morbidity and mortality in groups

**groups** **Condition **	**bortezomib** ** group**	**Other treatments group**	**P** **-value**
Heart failure	3 (15%)	3 (15%)	1.000
Diarrhea	3 (15%)	1 (5%)	0.605
Neuropathy	2 (10%)	---	0.487
Death	1 (5%)	3 (15%)	0.605
